# Overexpression of *BraLTP2*, a Lipid Transfer Protein of *Brassica napus*, Results in Increased Trichome Density and Altered Concentration of Secondary Metabolites

**DOI:** 10.3390/ijms19061733

**Published:** 2018-06-12

**Authors:** Nini Tian, Fang Liu, Pandi Wang, Xiaohong Yan, Hongfei Gao, Xinhua Zeng, Gang Wu

**Affiliations:** Key Laboratory of Oil Crop Biology of the Ministry of Agriculture, Oil Crops Research Institute, Chinese Academy of Agricultural Sciences, Wuhan 430062, China; tiannini93@163.com (N.T.); liufang03@caas.cn (F.L.); wangpandi@caas.cn (P.W.); yanxiaohong@caas.cn (X.Y.); gaohongfei@caas.cn (H.G.); zengxinhua@caas.cn (X.Z.)

**Keywords:** *BraLTP2*, overexpression, trichome development, secondary metabolites, antioxidant enzymes, *Brassica napus*

## Abstract

Plant non-specific lipid transfer proteins (nsLTPs) belong to a large multigene family that possesses complex physiological functions. Trichomes are present on the aerial surfaces of most plants and include both glandular secretory hairs and non-glandular hairs. In this study, *BraLTP2* was isolated from *Brassica rapa* (*B. rapa*) and its function was characterized in the important oilseed crop *Brassica napus* (*B. napus*). *B. rapa* lipid transfer protein 2 (*BraLTP2*) belongs to the little-known Y class of nsLTPs and encodes a predicted secretory protein. In Pro_BraLTP2_::GUS (β-glucuronidase) transgenic plants, strong GUS activity was observed in young leaves and roots, while low activity was observed in the anther. It is noteworthy that strong GUS activity was observed in trichomes of the first four leaves of 4-week-old and 8-week-old seedings, however, it disappeared in 12-week-old seedings. In transgenic plants expressing a BraLTP2::GFP (green fluorescent protein) fusion protein, GFP fluorescence localized in the extracellular space of epidermal cells and trichomes. Overexpression of *BraLTP2* in *B. napus* caused an increase in trichome number and altered the accumulation of secondary metabolites in leaves, including 43 upregulated secondary metabolites. Moreover, transgenic plants showed significantly increased activities of antioxidant enzymes. These results suggest that *BraLTP2*, a new *nsLTP* gene, may play a role in trichome development and the accumulation of secondary metabolites.

## 1. Introduction

Trichomes, which are derived from epidermal cells (outermost embryo’s cells), are specialized unicellular or multicellular structures that have various functions depending on the plant species and organ [1]. The criterion that is most often used to classify trichomes is whether they are glandular or not [2]. In the model plant *Arabidopsis thaliana* (*A. thaliana*, *Cruciferae*), only non-glandular trichomes are found, which are unicellular and can be either unbranched, or have two to five branches [3]. Trichomes represent the first barrier for overcoming pathogens and herbivorous arthropods because of their hairy physical properties [4].

Trichomes produce and accumulate secondary metabolites—such as flavonoids [5,6], phenylpropanoids [7], terpenoids [8], alkaloid [9], and defensive proteins [10]—With strong antifungal activity [11]; potential as natural pesticides [12]; and antirdical properties [13,14], such as protection against UV radiation [15,16].

Previous studies have reported that trichome gene expression sequence tags (*ESTs*) have been associated with resistance-related genes and biosynthetic pathways for secondary metabolites in mint (*Mentha piperita*) [17], basil (*Ocimum basilicum*) [7,18], alfalfa (*Medicago sativa*) [19], sweet wormwood (*Artemisia annua*) [20], hops (*Humulus lupulus*) [21], Greek sage (*Salvia fruticosa*) [22], tobacco (*Nicotiana tabacum*) [23], and tomato (*Lycopersicum esculentum*) [24]. It has been suggested that non-specific lipid transfer proteins (nsLTPs) are involved in the regulation of secondary metabolite biosynthesis, and have a role in resisting biological and abiotic stress [25,26,27].

In higher plants, nsLTPs are small, abundant, and basic secreted proteins [28,29], categorized into nine types (type I–IX) based on a genome-wide analysis of rice, wheat, and *A. thaliana* according to sequence similarity and intervals of eight-cysteine motif (8 CM) [30]. Type Y nsLTPs were first included in the nine nsLTP types, but because of the fact that their position was not well supported, they were excluded from the alignment [30]. The first plant lipid transfer protein was isolated from potato tuber [31]. Most *nsLTP* genes have been isolated and characterized in flowering plants (angiosperms), especially from major crops such as wheat (*Triticum aestivum*) [30], rice (*Oryza sativa*) [30], soybean (*Glycine max*) [32], Chinese cabbage (*Brassica rapa* (*B. rapa*)) [33], and maize [34]. nsLTPs have been implicated in complex physiological functions, such as abiotic stress resistance [35,36,37,38], pathogen defense [39,40], cutin and wax metabolism [25], sexual reproduction [41,42], and cell development [43,44]. In tobacco, it has been reported that nsLTPs were involved in secondary metabolisms [27]. The tobacco *NtLTP1* gene encodes a trichome-specific lipid transfer protein, reported to be required for the transfer of metabolites from trichomes, which affected the accumulation of cuticular metabolites [27].

In this study, we characterized the *B. rapa* lipid transfer protein 2 (*BraLTP2*), which is expressed in leaf epidermal cells and trichomes. Additionally, we examined the biological function of *BraLTP2* related to trichome development and secondary metabolites in *Brassica napus* (*B. napus*)*.* Until recently, all type Y *nsLTP* genes were poorly studied, with no known function, providing a good opportunity to explore new physiological functions of this family in processes such as trichome development, as shown herein. *BraLTP2* is the first type Y *nsLTP* family gene that has been found to affect the growth and development of epidermal trichomes and their effects on secondary metabolites. Our study will help broaden and deepen our understanding of the *nsLTP* gene function and will lay a foundation for the future application of *nsLTP* genes in *Brassica* breeding.

## 2. Results

### 2.1. Gene and Protein Sequence Characterization

**Figure 1:**
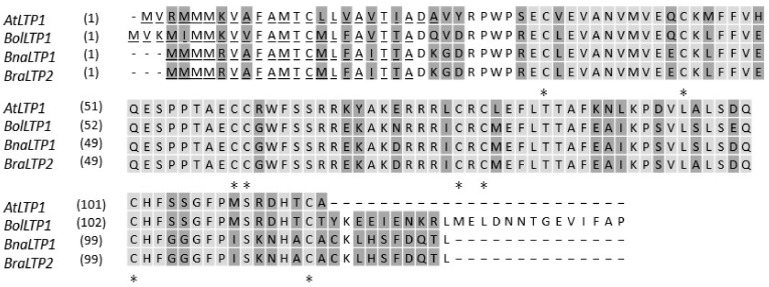
Analysis of the deduced amino acid sequences of *B. rapa* lipid transfer protein 2 *(BraLTP2)* with homologous sequences in other *Cruciferae*. Variable sites (dark grey), the nsLTP-like conserved 8 CM domain (light gray) with conserved cysteine residues (asterisks), and putative extracellular secretory signals (underlined) have been displayed. The sequences are from *Arabidopsis thaliana AtLTP1* (AT1g52415.1), *B. oleracea BolLTP1* (Bol019670), *B. napus BnaLTP1* (BnaAnng31360D), and *B. rapa BraLTP2* (Bra040156).

*BraLTP2* has an open reading frame of 375 bp, encoding a protein of 124 amino acid residues with a molecular mass of 14 kDa and a calculated isoelectric point of 7.5 (Figure 1). *BraLTP2* has 74% identity with *AtLTP1* from *Arabidopsis*, 99% identity with *BnaLTP1* from *B. napus*, and 85% identity with *BolLTP1* from *B. oleracea* (Figure 1)*.*

All species possessed high levels of sequence similarity in the nsLTP-like domain regions (Figure 1; light shaded amino acids 32 to 113 in *BraLTP2* and *BnaLTP1*, 36 to 116 in *BolLTP1*, and 35 to 115 in *AtLTP1*). A putative extracellular secretary signal (Figure 1; amino acids 1 to 24 in *Arabidopsis*, 1 to 23 in *B. oleracea*, and 1 to 20 in the other *Brassica* species) was 100% conserved in the *Brassica* species (Figure 1). Eighteen amino acid substitutions exist in the nsLTP-like domains between *AtLTP1* and the *Brassica* genes. The eight strictly conserved cysteine residues in all plant LTPs, which form four intrachain disulfide bridges, were also 100% conserved among all aligned sequences.

To obtain more information on the function of *BraLTP2*, we have predicted the *cis–*acting elements of the *BraLTP2* promoter using PlantCARE, and the results are listed in Table 1. The predicted *cis–*acting elements included typical light response elements, resistance response elements, phytohormone response elements, and protein binding sites of the *BraLTP2* promoter.

### 2.2. Spatiotemporal Expression of BraLTP2

**Figure 2:**
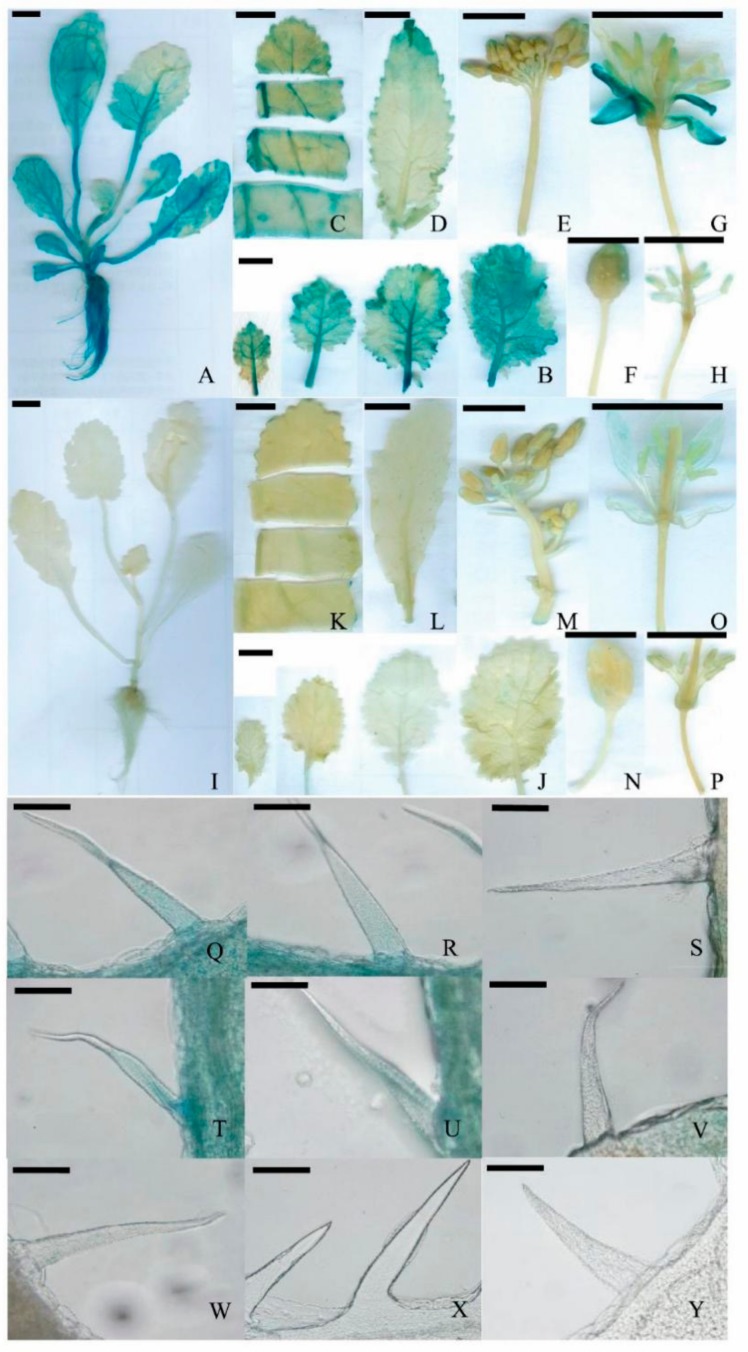
Histochemical β-glucuronidase (GUS) staining in tissues of Pro_BraLTP2_::GUS plants. (**A**): Four-week-old Pro_BraLTP2_::GUS seedling; (**B**): the first four lotus leaves from the apex of an 8-week-old Pro_BraLTP2_::GUS seedling; (**C**): the front part of the first four lotus leaves from the apex of a 12-week-old Pro_BraLTP2_::GUS seedling; (**D**,**E**,**F**): stem leaf and bud of a 15-week old plant during bud stage; (**G**,**H**): 18-week old Pro_BraLTP2_::GUS plant during flowering stage; (**I**–**P**): negative control groups in corresponding periods; (**Q**,**T**,**W**): Trichome of lotus leaves of 4-week old seedling; (**R**,**U**,**X**): trichome of lotus leaves of 8-week old seedling; (**S**,**V**,**Y**): trichome of lotus leaves of 12-week old seedling; (**Q**,**R**,**S**,**W**,**X**,**Y**): the first leaves; (**T**,**U**,**V**): the second leaves; (**Q**–**V**): trichome of Pro_BraLTP2_::GUS leaves; and (**W**–**Y**): trichome of negative controls. (**A**–**E**,**G**–**M**,**O**,**P**): Scale bars = 1 cm; (**F**,**N**): scale bars = 0.5 cm; and (**I**–**Y**): scale bars = 5 µm.

To study the tissue-specific expression of *BraLTP2*, the promoter region (1998 bp upstream of the ATG) was used to generate a translational fusion with the *β-glucuronidase* (*GUS*) reporter gene. Three transgenic Pro_BraLTP2_::GUS lines were analyzed in detail and showed a similar tissue-specific pattern (Figure 2). GUS expression was observed in the first four fully expanded leaves (Figure 2A,I) and their trichomes (Figure 2Q,T,W), and in stems and roots of 4-week-old seedlings (Figure 2A,I). For 8-week-old seedlings, strong GUS activity was observed in the vein of leaves (Figure 2B,J) and their trichomes (Figure 2R,U,X). For 12-week-old seedlings, strong GUS activity was observed in the vein of the leaf (Figure 2C,K), however, GUS activity in the trichomes all disappeared in the first four fully expanded leaves (Figure 2S,V,Y). During the bud stage (15-week-old plants), GUS expression was observed in the leaf tip and leaf edge (Figure 2D,L), as well as minor expression at the tip of the bud (Figure 2E,F,M,N). During the flowering stage (18-week-old plants), strong GUS expression was detected in sepals (Figure 2G,O,I), with minor expression observed at the tip of the stigma and stamen (Figure 2H,P).

### 2.3. Localization of BraLTP2::GFP (Green Fluorescent Protein) Fusion Protein

**Figure 3:**
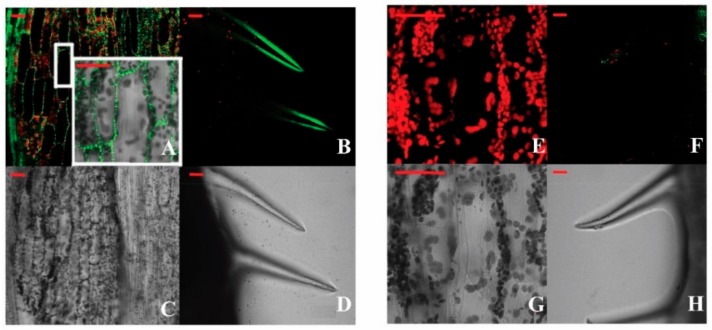
Localization of the fusion protein. (**A**–**D**): BraLTP2::GFP; and (**E**–**H**): negative controls. (**A**,**C**,**E**,**G**): *B. napus* petioles epidermis cells were transformed with construct and visualized with a fluorescence microscope; and (**B**,**D**,**F**,**H**): Trichomes on the leaf apex edge, visualized with a confocal laser scanning microscopy system. Scale bars = 50 µm.

A binary vector for the constitutive expression of a BraLTP2::GFP fusion protein was constructed and transformed into *B*. *napus* plants. The third fully expanded leaf from the apex of 4-week-old transgenic *B. napus* plants was visualized with a filter for the dual detection of GFP and chloroplast autofluorescence. The subcellular localization of *BraLTP2* was detected extracellularly (Figure 3A,C) in leaf epidermal cells of transgenic *B. napus*. Upon magnification, GFP fluorescence was mainly observed in the periphery of the cell walls (Figure 3A, white border). It is worth noting that GFP fluorescence was concentrated at the edge of the leaf trichomes (Figure 3B,D). No GFP fluorescence was observed in the negative control plants (Figure 3E,G); only weak chloroplast autofluorescence was detected in the negative control plant’s trichomes (Figure 3F).

### 2.4. Overexpressing BraLTP2 Increases Trichome Number

**Figure 4:**
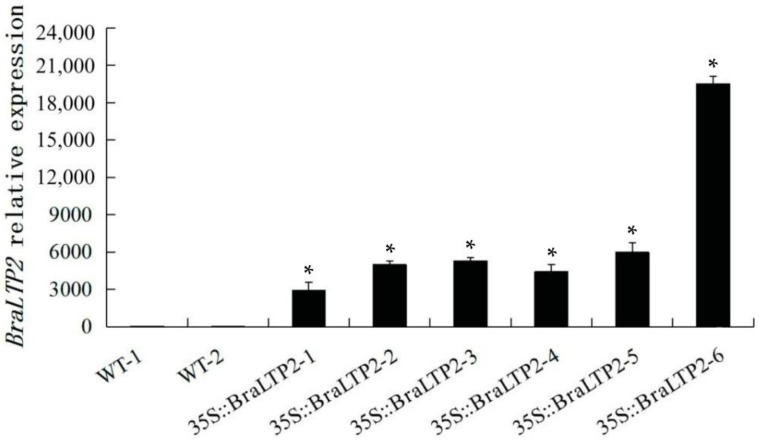
Analysis of *BraLTP2* mRNA levels in the wild type (WT) and *BraLTP2* overexpression lines. *BraLTP2* mRNA levels in 10-week-old wild type (WT) and *35S::BraLTP2* transgenic plants by real-time polymerase chain reaction (PCR). Actin was used as an internal loading control in the *y*-axis. Standard errors were derived from three biological repeated experiments for the expression levels of each T_0_ plant. * Statistically significant difference from wild type (* *p* < 0.05).

Six independent transformants of *B. napus* L. cultivar Zhongshuang 6 with the *35S::BraLTP2* construct were obtained. *BraLTP2* expression levels were 3000 to 19,000 times higher in the *BraLTP2* overexpressed lines relative to the wild type (WT), as determined by real-time polymerase chain reaction (PCR) (Figure 4). We chose two T_2_ *35S::BraLTP2* plants (*BraLTP2*-2 and *BraLTP2*-3) for further study because of their moderate expression and distinct phenotype observed on trichome density.

**Figure 5:**
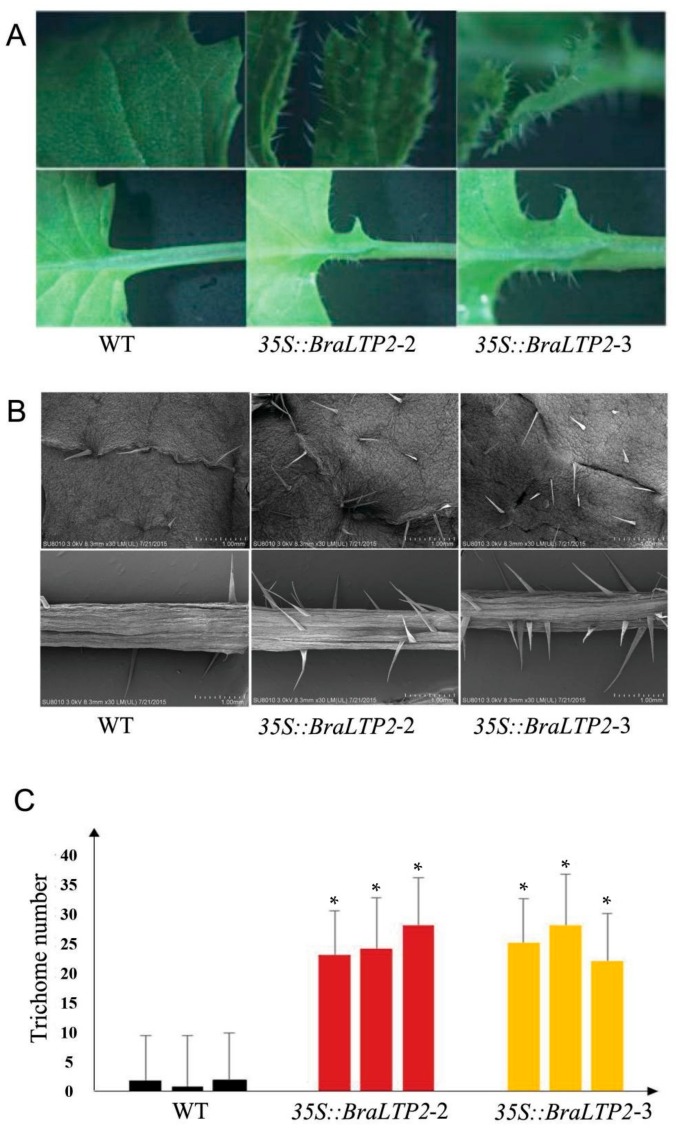
Phenotype identification, scanning electron microscopy (SEM) observation, and the number of epidermal trichome statistics. (**A**): Leaf epidermal trichomes imaged by light microscopy. (**B**): Leaf epidermal trichomes imaged by SEM. (**C**): Trichome number from 1 cm^2^ leaf area from apex edge. * Statistically significant difference from wild type (* *p* < 0.05).

The *35S::BraLTP2* plants show no difference in plant height and plant morphology when compared with WT plants, however, the transgenic plants exhibit a hairy leaf surface, which is not observed in WT plants. This phenotypic feature of the *35S::BraLTP2* plants was a result of densely distributed trichomes, visible on both abaxial/adaxial leaf surfaces and petiole, when compared with WT plants (Figure 5A).

Further observation by scanning electron microscopy (SEM) confirmed the increase in trichomes in the transgenic line when compared with the WT (Figure 5B). The number of trichomes in a 1 cm^2^ leaf area of the transgenic and WT plants was counted, and the number of trichomes in transgenic lines was approximately 20 times higher compared with that in the WT plants (Figure 5C).

### 2.5. Overexpressing BraLTP2 Affects Secondary Metabolites in B. napus

To investigate the metabolic changes in the *BraLTP2*-overexpressing leaves at the vegetative stage, widely targeted metabolic profiles were determined by the percentage change of every metabolite content between 4-week-old *BraLTP2*-overexpressing plants and WT plants at the five-leaf stage. A total of 494 metabolites were detected, of which 399 were known metabolites (Table S2) and 271 were known secondary metabolites (Table 2).

As we are more concerned about the composition and content of secondary metabolites, the 271 detected secondary metabolites have been classified into seven categories: flavones, phenylpropanoids, phenolamides, alkaloids (tryptamines), terpenoids, organic acids, and other metabolites. The exact amounts of each class of secondary metabolites are shown in Table 2.

The evaluation of the metabolites (shown in Table S1) by partial least squares discriminant analysis (PLS-DA) showed obvious clustering in each group and significant differences of secondary metabolite levels between the two groups (Figure S1). Eighty-nine differential metabolites, with the standard of variable important in projection (VIP) of ≥1 and fold change of ≥2 or ≤0.5, were identified by evaluation of all of the detected metabolites in Table S2 (Table S3), of which VIP ranged from 3.69 for *N*-hexosyl*-p*-coumaroyl serotonin to 1.2 for kaempferol-3-*O*-β-rutinoside (Nicotiflorin). Among the 89 differential metabolites, there were 73 differential secondary metabolites, of which 43 secondary metabolites were up regulated (VIP of ≥1 and fold change of ≥2) and 30 secondary metabolites were down regulated (VIP of ≥1 and fold change of ≤0.5), in *35S::BraLTP2* plants when compared with WT plants (Table 2).

**Figure 6:**
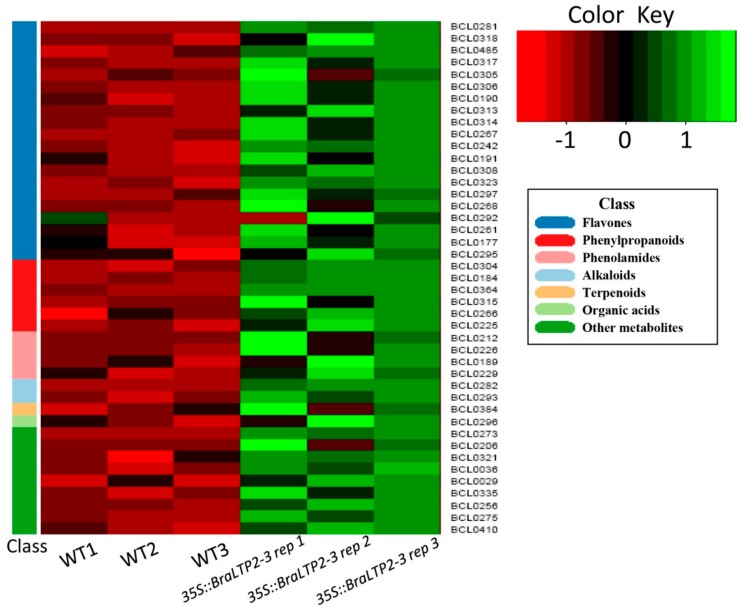
Differential classes of up-regulated secondary metabolites clustering heatmap. Seven classes of up-regulated secondary metabolites between *35S::BraLTP2*-3 rep 1–3 group and WT1–3 group in the clustering heatmap. The abscissa corresponds to the group number, the right ordinate corresponds to the secondary metabolites number, and the left ordinate of different color modules corresponds to the seven classes of metabolites. The color indicates abundance of changes in secondary metabolites from −1 to 1. Green corresponds to a higher correlation, the intensity of green is an indication of a higher content of secondary metabolites in the corresponding group.

Focusing on these 43 significantly up-regulated secondary metabolites, a hierarchical cluster analysis grouped these secondary metabolites clearly into two clusters based on their relative abundance (Figure 6). Secondary metabolites in the control group were detected in three biological repetitions (WT1, WT2, and WT3) and were indicated in relative low abundance (red). In contrast, greatly up-regulated secondary metabolites in the *BraLTP2-*overexpressed groups were detected in three biological repetitions (*35S::BraLTP2-3* rep 1, 35S::*BraLTP2-3* rep 2, and 35S::*BraLTP2-3* rep 3), and were indicated in relative high abundance (green). These results suggest high reproducibility within each group and a significant difference between WT and *35S::BraLTP2-3* groups (Figure 6). Table 3, Table 4, Table 5, Table 6 and Table 7 summarize these 43 significantly up-regulated secondary metabolites according to their classification of flavones, phenylpropanoids, phenolamides, alkaloids, terpenoids, organic acids, or other metabolites.

Specifically, the highest increase of secondary metabolites in *35S::BraLTP2* plants, compared with WT, was *N*-hexosyl-p-coumaroyl serotonin, a tryptamine, exhibiting a 105.77-fold increase (Table 6). IAA-Asp-N-Glc (indoles derivatives, Table 7), Kaempferide (flavone, Table 3), and N-feruloyl agmatine (phenolamides, Table 5) showed the second (57.09-fold), third (51.87-fold), and fourth (39.54-fold) highest increases, respectively.

Table 3 lists 20 flavones of *35::BraLTP2-*overexpressed lines with significant 2–52 times increased mass spectral signal values when compared with WT leaf samples, which was probably caused by overexpressing *BraLTP2*. Table 3 also lists the most important precursor up regulated in anthocyanin biosynthesis, such as cyanidin (3.00-fold changes) and cyanidin 3-*O*-glucoside (4.06-fold changes) in *35S::BraLTP2* plants, compared with WT plants. Table 4 lists the significant differences of phenylpropanoids metabolites, such as ferulic acid (5.72-fold changes), trans-Cinnamaldehyde (2.44-fold changes), and coumarin (2.27-fold changes).

### 2.6. Activities of Antioxidant Enzymes

**Figure 7:**
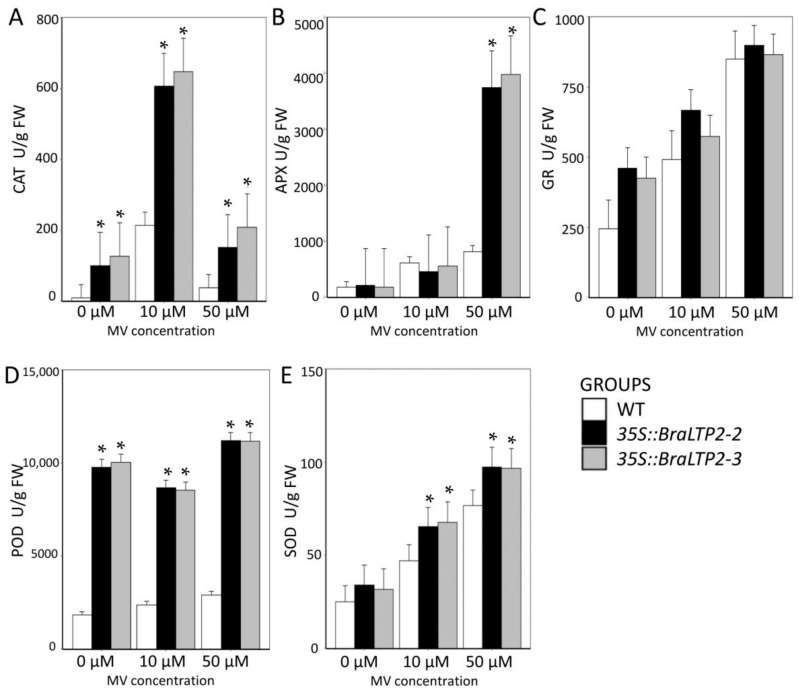
Changes in activities of antioxidant enzymes of WT, *35S::BraLTP2*-2, and *35S::BraLTP2*-3 leaves. The activity of antioxidant enzymes was determined after treatment with methyl viologen (MV) for four hours. * Statistically significant difference from wild type (* *p* < 0.05) in each different treatment. Experiments were performed in triplicate. (**A**): CAT—Catalase; (**B**): APX—Ascorbate peroxidase; (**C**): GR—Glutathione reductase; (**D**): POD—Peroxidase; and (**E**): SOD—Super oxide dimutese.

*BraLTP2*-overexpressed plants exhibited significantly increased levels of secondary metabolites. Kaempferide increased approximately 52 times, *N*-hexosyl-*p-*coumaroyl serotonin increased 105.7 times, and brevifolincarboxylic acid increased 4.9 times. Some researchers have indicated that kaempferide, *N*-hexosyl-*p-*coumaroyl serotonin, and brevifolincarboxylic acid all confer great antioxidation activity [45,46,47]. To confirm the role of oxidant resistance in *35S::BraLTP2* plants, we analyzed a series of antioxidant enzymes including catalase (CAT), ascorbate peroxidase (APX), glutathione reductase (GR), peroxidase (POD), and super oxide dimutese (SOD) after treatment with methyl viologen (MV), an oxidative stress inducer. Treatment with 10 µM and 50 µM MV resulted in significantly increased activity of CAT and SOD in *35S::BraLTP2* plants when compared with the WT. Treatment with 50 µM MV instead of 10 µM MV resulted in a significant increase of APX activity in *35S::BraLTP2* plants when compared with the WT. No significant increases were observed in GR activities of *35S::BraLTP2* plants after MV treatment and, interestingly, POD activity increased significantly in *BraLTP2*-overexpressed plants when compared with the WT, irrespective of treatment with MV (Figure 7, Student’s *t*-test, *p* < 0.05).

## 3. Discussion

### 3.1. Functional Characterization of BraLTP2

In this study, we have isolated and identified a *nsLTP* family gene from *Brassica rapa* accession Chiifu, which we named *BraLTP2*. The *nsLTP* family of genes are involved in a variety of physiological functions, however, research of *nsLTP* genes in *B. napus* is limited, and research of *nsLTP* genes related to epidermal trichome development and secondary metabolism has never been reported. In this study, we cloned and functionally analyzed a type Y *nsLTP* from *B. napus*.

*BraLTP2* has a 375-bp coding region with a homologous gene in the ‘C’ genome of *B. olercaea* and corresponding ‘A’ genome copies in the amphidiploid of the ‘AC’ genome of *B. napus*. The amino acid sequence similarity of *Arabidopsis*, *B. rapa*, *B. oleracea*, and *B. napus LTP2* copies is high and extends throughout the whole protein, with more variation in the *Arabidopsis LTP2* copy (Figure 1). The *B. napus* (*Brassica* AC genome) is an allotetraploid species resulting from a cross between *B. rapa* (A genome) and *B. oleracea* (C genome), so it is not surprising that the *BraLTP2* protein has a high identity to the LTP from *B. napus* and *B. oleracea* genomes [48]. According to previous reports, the configuration of the 8 CM domain and inter-cysteine amino acid residues places *BraLTP2* in type Y of the nsLTP large family, which, in *Arabidopsis*, is composed of uncharacterized proteins, including At1g52415, At1g64235, At4g08530, and At4g28395 [30]. The *BraLTP2* promoter contained a predicted MYB (v-myb avian myeloblastosis viral oncogene homolog) transcription factor binding site, or MBS for short (Table 1), indicating that *BraLTP2* may be regulated by upstream MYB transcription factors, which are involved in abiotic stress [49]. The predicted typical light response elements, resistance response elements, phytohormone response elements, and protein binding sites of the *BraLTP2* promoter suggested that *BraLTP2* may participate in resistance to biotic or abiotic reaction stress.

### 3.2. BraLTP2 Is Expressed in Leaf Epidermal Trichomes.

**Figure 8:**
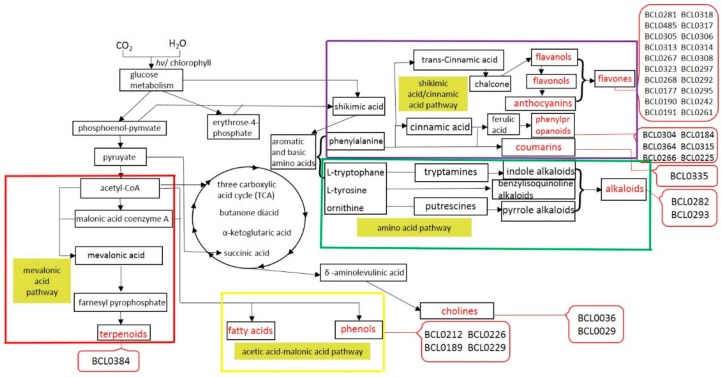
The relationship of different metabolic pathways. Important primary metabolic precursors and intermediate secondary metabolites are shown in black font, the final secondary metabolites are shown in red font. Pathways are boxed as follows: red box, mevalonic acid pathway; purple box, shikimic acid/cinnamic acid pathway; green box, amino acid pathway; yellow box, fatty acid pathway. The red callout boxes refer to the index number of up-regulated secondary metabolites, as detailed in Table S2.

In this study, *BraLTP2* promoter activity was found abundant in the vegetative parts of *B. napus* plants, mainly in the vein and petioles of young leaves (Figure 2). Interestingly, high *BraLTP2* promoter activity was observed in leaf trichomes of 4-week-old seedlings (Figure 2Q,T,W) and 8-week-old seedlings (Figure 2R,U,X), while it disappeared in 12-week-old plants (Figure 2S,V,Y). Likewise, trichome development initiates during the initial stages of leaf development [50], which indicates that *BraLTP2* is possibly involved in trichome initiation development in the early seeding stages.

In *Arabidopsis*, the *GL2* gene, which has the similar function to *BraLTP2*, is required for trichome development and affects trichome numbers, while its promoter regulated throughout the entire process of epidermal trichomes development [51,52]. Analysis of tobacco trichome expressed sequence tag (EST) libraries identified many trichome-specific genes and transcripts of several *LTPs* that accumulated specifically in trichomes [23]. Choi et al. [27] reported that tobacco *NtLTP1*, which is specifically expressed in long secretory glandular trichomes, plays a role in lipid secretion from trichome head cells and in resistance to aphid infestation. Yu et al. [53] have shown that squamosa promoter binding protein-like (*SPL*) genes temporally control the trichome distribution during flowering.

In this study, the cellular localization of the *BraLTP2* protein was examined in trichomes by the generation of transgenic plants expressing a BraLTP2::GFP fusion protein. GFP fluorescence was clearly visible at the periphery of epidermal cells including trichome cells (Figure 3A–D), indicating that the protein is excreted. Furthermore, *BraLTP2* contains a putative extracellular secretory signal (Figure 1), which is expected as *BraLTP2* belongs to the nsLTP family, most of which present an N-terminal secretory signal peptide and were detected extracellularly [44,54]. The morphology of the trichomes displayed in our research indicated that trichomes of *B. napus* are non-glandular trichomes, like *Arabidopsis* trichomes, as glandular trichomes normally have a large head with secretory cells. Our result indicated that the *BraLTP2* protein is secreted into the extracellular matrix of epidermal cells, including trichome cells.

More studies also provide evidence for an extracellular role of nsLTPs [55,56]. Lange et al. [17] have reported that in peppermint, LTPs are abundantly expressed in trichomes and have been proposed to transport lipid molecules to the periphery of the cell [17,57]. Several observations revealed that LTPs are secreted proteins; for example, an LTP, named PAPI protein, is secreted from aleurone layers into the incubation medium in *Hordeum vulgare* (barley) [58]. Two lipid transfer proteins were expressed entirely de novo in the tobacco leaf apoplast proteome [59] and in the secretome of the soybean xylem proteome [55].

### 3.3. Overexpressing BraLTP2 Leads to an Increase of Trichome Number

Trichome density of *35S::BraLTP2* plant leaves was significantly higher (*p* < 0.05) when compared with the WT (Figure 5). This phenotype resulted from the specific overexpression of *BraLTP2*, rather than from tissue culture mutation or vector insertion effects, because this phenotype was widespread in all lines. *BraLTP2* is the first nsLTP family member known to affect the development of trichomes exhibiting the hairy phenotype. However, there was no specific alteration on trichome morphology in *35S::BraLTP2* plants, suggesting that the *BraLTP2* gene might play a role in trichome formation, initiation, or development, and that the effect of *BraLTP2* overactivity was specifically to increase the trichome number.

Mutant analyses have implicated a role for trichome numbers in *Arabidopsis* [60,61], however, the *nsLTP* gene family has seldom been involved. In our study, we speculated that trichomes’ formation and density were promoted by up regulating the expression of *BraLTP2*.

There are two explanations that could support the above conclusion: (1) *BraLTP2* belongs to *nsLTP* gene family members, some of which had been reported to be involved in resistance to biotic and abiotic stress [37,62,63], cell growth development [43,64], and secondary metabolism [22,23,27]. *BraLTP2* has similar conserved domains and signal peptides to the *nsLTP* gene family (Figure 1). Therefore, it might show a similar gene function; (2) By analyzing the *BraLTP2* promoter, we found several cis-elements involved in biotic and abiotic stress (Table 1). We also found the protein binding site, including MBS, which is a MYB factor involved in drought induction (Table 1). Many studies have reported that MYB transcription factors are involved in epidermal trichome development and secondary metabolism; for example, *AtMYB23* regulates the trichome initiation of leaf edges in *Arabidopsis* [65]. The poplar gene, *PtaMYB186*, is a regulator of trichome initiation, and overexpression of *PtaMYB186* results in a fuzzy trichome phenotype [66]. In *Arabidopsis*, HIG1/MYB51 was shown to activate promoters of secondary metabolite–indolic glucosinolate biosynthetic genes, leading to increased accumulation of indolic glucosinolates [67]. We speculated that *BraLTP2* is regulated by a MYB transcription factor or other proteins with similar roles. Further investigation of the molecular mechanism of the *BraLTP2* action will shed more light on its function in trichome development and upstream regulation pathway.

### 3.4. Overexpressing BraLTP2 Alters Different Secondary Metabolites Produced by Different Biosynthesis Pathways.

It has been reported that nsLTP affects metabolites’ biosynthesis or secretion. For example, in *Arabidopsis*, *LTPG*, which encodes a glycosylphosphatidylinositol-anchored lipid transfer protein, has been reported to be required for the export of metabolites to the plant epidermal cells, which contributes either directly or indirectly to epidermal metabolites’ biosynthesis [25,26]. It has been reported that trichome development has a close relationship with increased metabolite levels, including anthocyanins, flavonoids, and phenylpropanoids [68,69]. In *Arabidopsis*, expression maps of leaf trichomes revealed high activities of anthocyanin and flavonoid pathways, indicative of the roles of trichomes in the biosynthesis of secondary metabolites and defense [68]. In our study, overexpressing *BraLTP2* led to the increase of the trichome number. Therefore, we explored the metabolite changes in transgenic and WT *B. napus* plants.

Secondary metabolites from the leaf of the transgenic lines and the WT were analyzed by liquid chromatography tandem mass spectrometry (LC-MS). The metabolic network involved in different secondary metabolites’ biosynthesis is well known [70]. We detected 43 up-regulated secondary metabolites, of which 36 are shown in the metabolic network of Figure 8, which is an overview map of key up-regulated secondary metabolites, synthesized by the different biosynthetic pathways, in *35S::BraLTP2* plants compared with the WT.

The first pathway (Figure 8) is the acetic acid-malonate acid (AA-MA) pathway; the precursors, acetyl-CoA and malonic acid coenzyme A, synthesize fatty acids, and acetyl-CoA can form phenols through condensation reactions [71,72]. In this biosynthetic pathway, we have detected up regulation of BCL0212 (*N*-feruloyl agmatine), BCL0226 (*N*-Sinapoyl agmatine), BCL0189 (*N*-Sinapoyl agmatineand), and BCL0229 (*N*-Feruloyl spermidine) (Table 5).

The second pathway (Figure 8) is the mevalonic acid pathway; it produces terpenoid compounds via certain biosynthetic reactions with the precursor of mevalonic acid (MVA) [70]. BCL0384 (phytocassane C), showing 2.39-fold overexpression when compared with the WT (Table 6), is a precursor for the synthesis of diterpenes, which are diterpenoids with a structure based on the isocopalane (Tetradecahydro-1,1,4a,7,8,8a-hexamethylphenanthrene) or the 15,16-epoxyisocopalane skeleton [73].

The third pathway (Figure 8) is the shikimic acid/cinnamic acid pathway [70,74]; the aromatic amino acid, phenylalanine, is a precursors to synthesize into phenylpropanoid compounds, as well as flavanols, flavonols, and anthocyanins [70,74]. It is well known that flavones are synthesized via the phenylpropanoid pathway and can be divided into six major subgroups in plants, including flavanones, flavonoids, flavonols, flavan-3-ols, anthocyanins, and isoflavones [75,76].

The fourth pathway (Figure 8) is the amino acid pathway; alkaloids are synthesized by three pathways with three amino acids as precursors, l-tryptophane, l-tyrosine, and ornithine. Table 6 lists the significant difference of indole alkaloids in the l-tryptophane amino acid pathway. *N-*Hexosyl-*p-*coumaroyl serotonin (105.77-fold increase), which has the most significant difference of secondary metabolites, is a kind of tryptamines.

The fifth pathway, acetyl-CoA, can also take part in the tricarboxylic acid (TCA) cycle, then form the δ-aminolevulinic acid, and finally form cholines. Table 7 has listed the significant difference reactants, such as *O*-phosphocholine (3.54-fold increase) and sn-glycero-3-phosphocho (2.41-fold increase).

Our results indicate that *BraLTP2* may participate in secondary metabolites’ biosynthesis or storage in *B. napus* leaves. However, further investigation is necessary to determine how *BraLTP2* affected these secondary metabolite biosynthetic pathways.

### 3.5. Overexpressing BraLTP2 Demonstrates Enhanced Activities of the Main Antioxidant Enzymes

Plant stress resistance is responsible for the tolerance of plants to adverse environments; a product of evolution. Plants under environmental stress can prevent, reduce, or repair damage caused by adversity through metabolic reactions to maintain normal physiological activities [77,78]. It has been reported that secondary metabolites such as flavones and phenylpropanoids play an important role in oxidation tolerance and UV-B (with a wavelength of 280–315nm) resistance of plants [45,46,47,79]. The level of the flavone kaempferide (Table 3), which confers antioxidation activity [47], was 52-fold higher in the *35S::BraLTP2* plants. Likewise, *N*-hexosyl-*p-*coumaroyl serotonin, which increased 105.7-fold over the WT (Table 6), has an antioxidant role in rice leaves [45,46]. Ferulic acid, with an increase of 5.7-fold (Table 4), plays a role in shielding UV-B radiation [80]. Brevifolincarboxylic acid, which has a 4.9-fold increase (Table 4), has also been shown to act as an antioxidant [81,82].

The *35S::BraLTP2* plants have increased activities of the main antioxidant enzymes such as CAT, APX, POD, and SOD (Figure 7), which are responsible for alleviating or preventing MV-induced oxidative injury such as the reactive oxygen species (ROS) formation in plants [83]. SOD forms the first line of defense against ROS under stress [84], reducing superoxide (O_2_^−^) into hydrogen peroxide (H_2_O_2_)_._ H_2_O_2_ can be metabolized into oxygen and water by CAT and POD. H_2_O_2_ is also restricted by the ascorbate–glutathione (ASH–GSH) cycle, where APX uses ASH as a hydrogen donor and GR catalyzes the NADPH-dependent reduction of oxidized glutathione (GSSG) to reduced GSH [85]. In our study, in *35S::BraLTP2* transgenic plants leaves, the CAT, GR, POD, and SOD activities under MV treatment were higher than those in the WT. Under MV treatment, CAT, POD, and SOD activity showed a significant (*p* < 0.05) increase in *35S::BraLTP2* transgenic leaves when compared with WT leaves (Figure 7), but APX activity significantly (*p* < 0.05) increased only under high MV concentrations (50 µM). GR had no significant increase under any MV concentration. With 10 µM MV treatments, CAT and POD would work to remove ROS, and as APX and GR are part of the ASH-GSH cycle that scavenges ROS [86], no increase in APX or GR activities would be required to maintain safe levels of ROS. Enhanced activities of antioxidant enzymes play a role in the oxidation pathway, thereby improving the oxidative stress tolerance of *35S::BraLTP2* plants. There are also some reports that have proved that the activities of these five antioxidant enzymes were increased by heavy metal (cadmium) stress in wheat, tobacco, and *Miscanthus* spp*.* [87,88,89].

## 4. Materials and Methods

### 4.1. Plant Material

The plants seeds were sterilized and germinated on sterile Murashige & Skoog medium. The germinated seedlings or rooted transgenic plants were transferred from Murashige & Skoog media into pots containing a mixture of peat moss (PINDSTRUP, Ryomgaard, Danmark) and field soil (3:1), and maintained in a growth chamber at 18 °C ± 2 °C with a 16 h light and 8 h dark photoperiod, at a light intensity of 44 µmol m^−2^ s^−1^ and relative humidity of 60–90%.

### 4.2. Gene, Protein, and Promoter Sequence Analysis

*BraLTP2* was aligned to homologous amino acid sequences from several Cruciferae species, including *Arabidopsis*, *B. napus*, and *Brassica oleracea* (*B. oleracea*), using Align X multiple sequence alignment software (Vector NTI Advance 11.0, 2008 Invitrogen Corporation, Carlsbad, CA, USA). The homology search was conducted using TAIR (http://www.arabidopsis.org/) and BRAD (http://brassicadb.org/brad/). Conserved domains were identified using CDD (http://www.ncbi.nlm.nih.gov/cdd/) and the signal peptide was determined by SignalP (http://www.cbs.dtu.dk/services/SignalP/) [64]. The predicted protein information was conducted using EXPASY (http://www.expasy.org/resources; Gigolashvili et al., 2007). The core promoter region and upstream cis-acting elements were predicted by the promoter prediction software, PlantCARE (http://bioinformatics.psb.ugent.be/webtools/plantcare/html/) (Universiteit Gent, Gent, Belgium).

### 4.3. Vector Construction and Genetic Transformation

#### 4.3.1. Gene Cloning and Vector Construction

Genomic DNA was isolated from the *B. rapa* accession Chiifu using primers designed against the published *B. rapa* sequence Bra040156 (http://brassicadb.org/brad/index.php) [90], and the *BraLTP2* DNA fragment was obtained via PCR, using the forward (*BraLTP2*-F) and reverse (*BraLTP2*-R) primers containing 5’ restriction enzyme sites for *Sac*I and *BamH*I, respectively (Table S1). PCR was carried out in 50 µL, with 50 ng DNA, 0.2 mM dNTPs, 0.3 µM of each primer, 1.0 U KOD plus Taq (ToYoBo, Osaka, Japan), 1× KOD plus Taq buffer (ToYoBo, Osaka, Japan), and 1.0 mM MgSO_4_ (ToYoBo, Osaka, Japan). The conditions were as follows: 94 °C for 3 min, 30 cycles at 94 °C for 30 s, 56 °C for 30 s, and 68 °C for 30 s. The resulting 477 bp amplification product was digested by *Sac*I (Thermo Scientific™ Code: ER1131, Rochester, NY, USA) and *BamH*I (Thermo Scientific™ Code: ER0051), and subcloned into the pMD^®^18-T cloning vector (Takara, Tokyo, Japan). To construct the *BraLTP2* overexpression plasmid, the cloning vector harboring *BraLTP2* was transferred to the pBI121S destination vector between the CaMV 35S promoter and a terminal poly A sequence [64]. The integrity of the construct was confirmed by sequencing.

A 1998-bp upstream DNA fragment containing the *BraLTP2* promoter was amplified with the forward primer Pro_BraLTP2_-F and reverse primer Pro_BraLTP2_-R (Table S1). The PCR product was digested with *Pst*I (Thermo Scientific™ Code: ER0611) and *BamH*I, and then cloned into the pMD^®^18-T cloning vector (Takara). The destination fragment was cleaved from the cloning vector and ligated into the destination vector pDX2181G using a T4 DNA ligase. The integrity of the construct was confirmed by sequencing and PCR analysis.

To determine the subcellular localization of *BraLTP2* in plant cells, we created a BraLTP2::GFP fusion construct. For *BraLTP2* amplification, the forward primer BraLTP2::GFP-F and reverse primer BraLTP2::GFP-R (containing 5’ restriction enzyme sites for *Sac I*) were used (Table S1). For *GFP* amplification, the forward primer GFP-F (containing 5’ restriction enzyme sites for *BamH*I) and reverse primer GFP-R were used (Table S1). The target *BraLTP2* and *GFP* genes were combined into a fusion gene in the pBI121S vector under the control of the CaMV 35S promoter.

#### 4.3.2. Genetic Transformation

Etiolated hypocotyls of *B*. *napus* cv. Zhongshuang 6, an elite Chinese cultivar from China, were transformed by *Agrobacterium tumefaciens* strain GV3101 (Weidi Biotechnology Co., Ltd, Shanghai, China) and regenerated, as described by Liu et al. (2014) [64].

### 4.4. Real-Time PCR Analysis

Total RNA was isolated from the fourth fully expanded leaf from the apex for each 10-week-old T_0_ generation *B. napus* plant using the TIANGEN RNAprep Pure Plant Kit (DP 432, TIANGEN, Beijing, China), according to the manufacturer’s instructions. Reverse-transcription reactions were carried out to synthesize the first-strand cDNAs from DNaseI-treated total RNA using a TIANGEN FastQuant RT Kit (with gDNase) (KR106, TIANGEN, Beijing, China), according to the manufacturer’s instructions. The cDNA was used as a template for PCR amplification analysis, and the reaction and procedure followed the manufacturer’s instructions, as above, in four replicates for each cDNA sample. PCR primers (Rt-PCR-F and Rt-PCR-R) and TaqMan probes (Rt-PCR-P) were designed based on the *BraLTP2* cDNA sequences (Table S1). Specific primers (*Actin*-F and *Actin*-R) and TaqMan probes (*Actin*-P) for the *B. napus Actin* gene (GenBank accession number: AF111812.1) were used as an internal control (Table S1). Real-time PCR was performed in an optical 96-well plate with a Bio-Rad CFX96 Real-Time System (C1000 Thermal Cycler) (Applied Biosystems, Hercules, CA, USA).

### 4.5. Histochemical Analysis of GUS Expression

The Pro_BraLTP2_::GUS clone in DX2181G was used to transform *A. tumefaciens* GV3101, and subsequently *B. napus*. *B. napus* transformants were selected with hygromycin B (25 mg/L) and verified by PCR analysis. Histochemical GUS staining of the T_2_ generation transgenic plants harboring the Pro_BraLTP2_::GUS construct were conducted, as described by Jefferson et al. [91], using negative transgenic seedlings as controls. Briefly, young seedlings of transgenic lines expressing Pro_BraLTP2_::GUS were incubated for 12 h with reaction buffer solution (50 mM Na_2_HPO_4_–NaH_2_PO_4_, pH 7.0, 0.5 mM K_3_Fe(CN)_6_, 0.5 mM K_4_Fe(CN)_6_, containing 2 mM 5-bromo-4-chloro-3-indolyl-b-d-glucuronic acid as a substrate). Tissue was destained with 70, 80, and 100% ethanol, trichomes were observed and photographed under an optical microscope (Olympus IX71, Melbourne, Australia), and other tissues were examined and photographed by a scanner (HP Scanjet G4050, Hewlett Packard, Beijing, China).

### 4.6. Microscopic Observation of the BraLTP2::GFP Fusion Protein

The BraLTP2::GFP fusion construct was transformed into *B. napus* plants mediated by *A. tumefaciens* strain GV3101, as described previously, using negative transgenic seedlings as controls. Leaf epidermal cells of T_2_ generation plants were analyzed to determine the location of the fusion protein, utilizing a confocal laser-scanning microscopy system (LSM) (Nikon A1, Tokyo, Japan) after treatment of light avoidance at 25 °C for 1 h. Fluorophores were excited using an argon laser at 488 nm (GFP), and bright-field images were collected using a transmitted light detector.

### 4.7. Trichome Observation

To compare the phenotypes between transgenic plants and the wild type (WT) plants, the second fully expanded leaves of 4-week-old T_2_ seedlings were observed by optical microscopy (Olympus SZX16, Melbourne, Australia). Furthermore, to observe the morphology and density of trichomes, young fresh leaves and stems of 4-week-old T_2_ seedlings were observed using scanning electron microscopy (SEM) (Hitachi SU8010, Tokyo, Japan). Before observation, the samples were fixed with glutaraldehyde and washed in cacodylate buffer, and then dehydrated in ethanol, dried, and coated with a film of gold [92]. To count the number of trichomes, an abaxial leaf area of 1 cm^2^ from the second fully expanded leaf from the apex for each 4-week-old plant was used for microscopic analysis and measurement. The leaves from three randomly selected plants of each transgenic line, along with the WT, were observed.

### 4.8. Analysis of Secondary Metabolites from Transgenic Plants by Liquid Chromatography Tandem Mass Spectrometry (LC-MS)

The third fully expanded leaves from the apex for T_2_ generation *BraLTP2*-overexpressing and WT seedlings at the five-leaf stage (4-week-old) were harvested for secondary metabolites analysis. Three independent biological repeats were performed, with five sample mixes for each repeat in order to reduce the metabolite differences caused by the environment and individuals [93,94,95]. These samples were frozen in liquid nitrogen, followed by freezing/drying (Scientz-100F, Ningbo, China), and ground for 1.5 min at 30 Hz using a grinding apparatus (MM 400, Retsch, Shanghai, China). The ground powder (100 mg) was extracted, separated, and analyzed using liquid chromatography tandem mass spectrometry (LC-MS) to identify and quantify the metabolites present, as previously described [93]. A previously described, the relative quantification method was used to analyze the samples [94]. Metabolite (m-trait) data were log2 transformed for statistical analysis to improve normality. The m-trait data of the association panel are the mean of the two biological sample sets for the LC-MS data, as follows: P*_m_*, l = 1/2(P*_m_*,l,1 + P*_m_*,l,2), where P*_m_*, l represents the m-trait data for metabolite m (m = 1, 2, 3, ..., 494 in leaf), and P*_m_*,l,1 and P*_m_*,l,2 are the normalized metabolite levels determined in the two biological sample sets, respectively. Principal component analysis plots were used to infer the difference between the two samples. Principal component analysis of the metabolites was performed using the software SIMCA-P with default settings.

### 4.9. Determination of Anti-Oxidant Enzymes Activities

One hundred milligrams fresh weight of the second fully expanded leaves from the apex for 2-week-old T_2_ generation *BraLTP2*-overexpressed transgenic and WT plants were harvested, with three technical replicates and three biological replicates. The samples were soaked in an aqueous 10 µM and 50 µM methyl viologen (MV, Sigma, Shanghai, China) [96] solution for 4 h, with distilled water treatment as a control. The leaves were then frozen in liquid nitrogen and ground at 4 °C in a mortar and pestle, with 1 ml extraction buffer (50 mM phosphate buffer (pH 7.8), containing 0.1 mM EDTA, 0.5% (*w*/*v*) Triton-100 and 2% polyvinyl pyrrolidone (PVP)), and centrifuged at 8000× *g* at 4 °C for 10 min (catalase (CAT), peroxidase (POD), super oxide dimutese (SOD) activity); at 8000× *g* at 4 °C for 15 min (glutathione reductase (GR) activity); and at 13,000× *g* at 4 °C for 20 min (ascorbate peroxidase (APX) activity). The supernatants were analyzed for enzyme activity using commercial kits, according to the manufacturer’s instructions (Jiangsu Keming Biotechnology Institute, Suzhou, China).

## 5. Conclusions

*BraLTP2* is a new gene from *B. rapa*, its function, as well as that of its homologous gene in *Arabidopsis*, has not been previously identified. We have demonstrated that overexpressing *BraLTP2* led to an increased number in leaf epidermal trichomes, suggesting *BraLTP2* might play a role in trichome development. We observed an increase in secondary metabolites, such as flavones, phenylpropanoids, phenolamides, alkaloids, terpenoids, organic acids, and other metabolites, in *35::BraLTP2-*overexpressed *B. napus* leaves when compared with those of WT, which may play a role in resistance to oxidation stress. Additionally, we have demonstrated increased activities of CAT, POD, and SOD under MV treatment, also contributing to oxidation tolerance in *B. napus*. *B. napus* trichomes were observed as non-glandular trichomes in our study, which may have the capacity to synthesize or store a wide array of metabolites. However, how much the increase of trichomes contributed to the increase of metabolites in this study still needs to be made clear.

## Figures and Tables

**Table 1:** The putative *cis*-acting regulatory elements in the promoter of *BraLTP2* gene.

Component Name	Organism	Position in (+) Strand		Sequence	Function
		Start	End		
ABRE	*Arabidopsis thaliana*	–1184 –254 –166	–1179 –250 –160	CACGTGACGTGCACGCGG	*cis–*acting element involved in the abscisic acid responsiveness
ARE	*Zea mays*	–661 –546	–655 –541	TAACGTATGGTTT	*cis–*acting regulatory element essential for the anaerobic induction
P-Box	*Petroselinum crispum*	–975	–970	TTGACC	Gibberellin-responsive element
CGTCA-motif	*Hordeum vulgare*	–213	–207	CCTTTTG	*cis–*acting regulatory element involved in the Methyl jasmonate-responsiveness
DRE	*Arabidopsis thaliana*	–1141	–1134	TGGCCGAC	Regulatory element involved in cold- and dehydration-responsiveness
G-Box	*Antirrhinum majus*	–778	–773	CACGTT	*cis–*acting regulatory element involved in light responsiveness
MBS	*Arabidopsis thaliana*	–1090	–1085	CAACTG	MYB binding site involved in drought-inducibility
MYB	*Arabidopsis thaliana*	–109 –67	–104 –62	CTGTTA	MYB binding sites

**Table 2:** Seven main categories of the secondary metabolite in the wild type (WT) and *35::BraLTP2-*overexpressed lines.

Class	Number of Compounds Detected	Number of Compounds with Significant Quantitative Differences	Number of Compounds with Higher Concentration in *35::BraLTP2*	Number of Compounds with Lower Concentration in *35::BraLTP2*
Flavonoids	55	21	20	1
Phenylpropanoids	32	10	6	4
Phenolamides	19	13	4	9
Alkaloids	24	8	2	6
Terpenoids	10	2	1	1
Organic acids	34	2	1	1
Other metabolites	97	17	9	8
Total	271	73	43	30

**Table 3:** Difference of flavones in the wild type and *35::BraLTP2-*overexpressed lines. VIP—variable important in projection.

No. ^a^	Flavones	WT ^b^	*BraLTP2* ^b^	Fold Change	VIP
BCL0281	Kaempferide	6.07 × 10^2^	3.15 × 10^4^	51.87	3.35
BCL0318	Prunin	1.27 × 10^3^	7.03 × 10^3^	5.56	2.11
BCL0485	Kaempferol	1.03 × 10^4^	5.59 × 10^4^	5.43	2.22
BCL0317	Chrysoeriol *O*-hexoside	2.02 × 10^3^	1.00 × 10^4^	4.95	2.10
BCL0305	Kaempferol 3-*O*-glucoside (Astragalin)	2.00 × 10^4^	9.20 × 10^4^	4.60	1.81
BCL0306	Luteolin 5-*O*-hexoside	4.14 × 10^4^	1.70 × 10^5^	4.10	1.98
BCL0313	Naringenin 7-*O*-glucoside	5.65 × 10^3^	2.08 × 10^4^	3.68	1.87
BCL0314	Apigenin 7-*O*-glucoside (Cosmosiin)	2.66 × 10^3^	9.35 × 10^3^	3.51	1.84
BCL0267	Luteolin *O*-hexosyl-*O*-hexoside	2.37 × 10^4^	7.42 × 10^4^	3.13	1.75
BCL0308	Apigenin 7-*O*-glucoside	5.35 × 10^3^	1.54 × 10^4^	2.87	1.72
BCL0323	Naringenin *O*-malonylhexoside	1.77 × 10^4^	5.03 × 10^4^	2.84	1.72
BCL0297	3’,4’,5’-Dihydrotricetin *O*-hexosyl-*O*-hexoside	3.97 × 10^3^	1.04 × 10^4^	2.62	1.59
BCL0268	Quercetin *O*-hexoside	1.01 × 10^4^	2.59 × 10^4^	2.57	1.49
BCL0292	Kaempferol 3-*O*-β -rutinoside (Nicotiflorin)	5.36 × 10^3^	1.36 × 10^4^	2.54	1.20
BCL0177	Luteolin-3’,7-di-*O*-glucoside	5.71 × 10^6^	1.36 × 10^7^	2.38	1.51
BCL0295	3,4,2’,4’,6’-Pentamethoxychalcone	1.31 × 10^4^	2.62 × 10^4^	2.00	1.27
BCL0190	Cyanidin 3-*O*-glucoside (Anthocyanins)	1.54 × 10^6^	6.24 × 10^6^	4.06	1.96
BCL0242	Cyanidin (Anthocyanins)	4.87 × 10^3^	1.46 × 10^4^	3.00	1.76
BCL0191	Delphinidin *O*-hexoside (Anthocyanins)	5.52 × 10^4^	1.65 × 10^5^	2.98	1.69
BCL0261	Rosinidin *O*-hexoside (Anthocyanins)	3.40 × 10^3^	8.49 × 10^3^	2.50	1.52

Note: ^a^ The No. is the secondary metabolite number in Table S1; and ^b^ The average value is shown in the table. The value means mass spectral signal response value.

**Table 4:** Difference of phenylpropanoids in the wild type and *35::BraLTP2*-overexpressed lines.

No.	Phenylpropanoids	WT	*BraLTP2*	Fold Change	VIP
BCL0304	Ferulic acid	2.60 × 10^3^	1.49 × 10^4^	5.7	2.24
BCL0184	Brevifolincarboxylic acid	1.61 × 10^4^	7.94 × 10^4^	4.93	2.13
BCL0364	Geranyl acetate	1.86 × 10^4^	7.48 × 10^4^	4.03	2.00
BCL0315	*trans*-Cinnamaldehyde	4.57 × 10^3^	1.11 × 10^4^	2.44	1.50
BCL0266	Syringic acid	2.00 × 10^3^	4.89 × 10^3^	2.44	1.52
BCL0225	Coumarin	9.77 × 10^6^	3.22 × 10^6^	2.27	1.48

**Table 5:** Difference of phenolamides in the wild type and *35::BraLTP2-*overexpressed lines.

No.	Phenolamides	WT	*BraLTP2*	Fold Change	VIP
BCL0212	*N*-Feruloyl agmatine	4.51 × 10^3^	1.78 × 10^5^	39.54	3.13
BCL0226	*N*-Sinapoyl agmatine	2.71 × 10^3^	1.92 × 10^4^	7.08	2.26
BCL0189	*N*-Coumaroyl agmatine	2.02 × 10^3^	8.02 × 10^3^	3.97	1.82
BCL0229	*N*-Feruloyl spermidine	8.84 × 10^4^	1.79 × 10^5^	2.03	1.34

**Table 6:** Difference of alkaloids, terpenoids, and organic acids in the wild type and *35::BraLTP2*-overexpressed lines.

No.	Alkaloids, Terpenoids, and Organic acids		WT	*BraLTP2*	Fold Change	VIP
BCL0282	Alkaloids (Tryptamines)	*N*-Nexosyl-*p-*coumaroyl serotonin	1.56 × 10^3^	1.65 × 10^5^	105.77	3.69
BCL0293		5-Methoxy-*N*,*N*-dimethyltryptamine	5.36 × 10^4^	1.85 × 10^5^	3.45	1.85
BCL0384	Terpenoids	Phytocassane C	6.71 × 10^3^	1.60 × 10^4^	2.39	1.32
BCL0296	Organic acids	m-Anisic-acid	7.44 × 10^3^	1.86 × 10^4^	2.83	1.57

**Table 7:** Difference of other metabolites in the wild type and *35::BraLTP2-*overexpressed lines.

No.	Other Metabolites		WT	*BraLTP2*	Fold Change	VIP
BCL0273	Indoles and its derivatives	IAA-Asp-N-Glc	7.41 × 10^2^	4.23 × 10^4^	57.09	3.38
BCL0321		1-Methoxyindole-3-carbaldehyde	7.44 × 10^3^	1.86 × 10^4^	2.50	1.56
BCL0036	Cholines	*O*-Phosphocholine	9.49 × 10^3^	3.36 × 10^4^	3.54	1.88
BCL0029		sn-Glycero-3-phosphocho	8.99 × 10^5^	2.17 × 10^6^	2.41	1.52
BCL0335	Coumarins and its derivatives	4-Methylumbelliferone	1.49 × 10^4^	3.51 × 10^4^	2.35	1.26
BCL0206	Others	*N*-α-Benzenolarginine ethylester	9.01 × 10^2^	1.15 × 10^4^	12.79	2.50
BCL0256		Bergamottin	6.23 × 10^5^	3.16 × 10^6^	5.08	2.14
BCL0275		1-(3,4-Dichlorophenyl)-3-methylurea	1.89 × 10^5^	8.77 × 10^5^	4.63	2.08
BCL0410		2-Amino-9-methyl-4-octadecene-1,3,8-triol	4.80 × 10^3^	1.12 × 10^4^	2.33	1.49

## Supplementary Materials

Supplementary materials can be found at http://www.mdpi.com/1422-0067/19/6/1733/s1.
