# Supplementary material for: Overexpression of BraLTP2, a Lipid Transfer Protein of Brassica napus, Results in Increased Trichome Density and Altered Concentration of Secondary Metabolites
Source: Int J Mol Sci. 2018 Jun 12;19(6):1733. doi: 10.3390/ijms19061733 (PMC6032385; doi:10.3390/ijms19061733)
Supplement: Supplementary file 1 [file ijms-19-01733-s001.zip › ijms-308616-Figure S1.pdf]

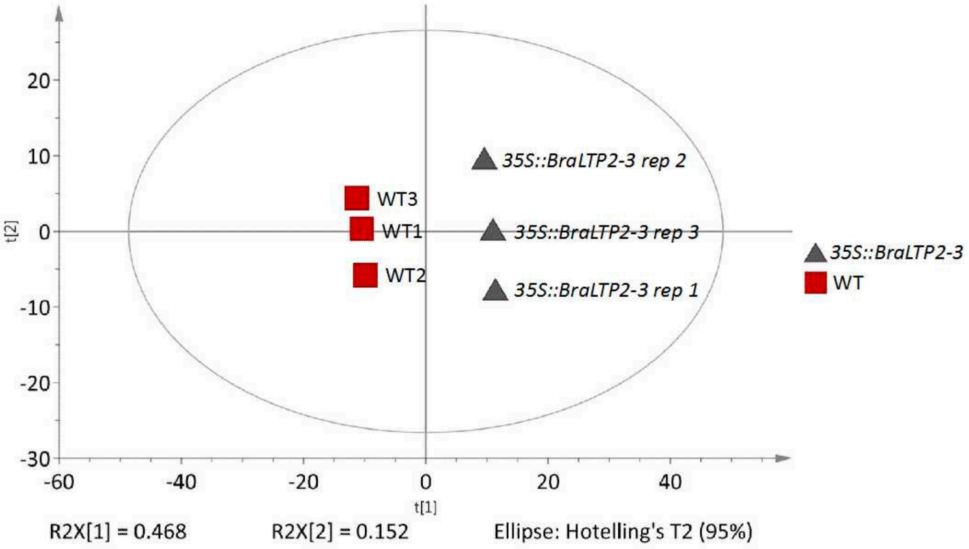

Figure S1: PLS-DA analysis of two groups of mass spectrometry data. Evaluation of the metabolites shown in Supplemental Table 1 by Partial Least Squares Discriminant Analysis (PLS-DA) showed obvious clustering in each group and significant differences of secondary metabolite levels between the two groups (Supplementary Figure 1). Sample aggregation or dispersion, reflected repeatability of samples and similarity of metabolic profiles between sample groups. Three samples of each group clustered together, showed high similarity among samples within groups. The PLS-DA data also showed significant separation between the two groups, indicating distinct differences of metabolites between overexpressing *BraLTP2* plants and WT plants.
